# An organics-forward approach to searching for life on Mars

**DOI:** 10.1038/s41467-026-72266-2

**Published:** 2026-05-11

**Authors:** D. K. Buckner, A. Mojarro

**Affiliations:** 1 NASA Postdoctoral Program, Oak Ridge Associated Universities, Oak Ridge, TN USA; 2 Solar System Exploration Division, NASA Goddard Space Flight Center (GSFC), Greenbelt, MD USA; 3 CRESST II, NASA GSFC, Greenbelt, MD USA

**Keywords:** Astrobiology, Astrobiology

## Abstract

The potential for life on Mars has intrigued humanity for centuries. An organics-forward approach to sample return and in situ analyses provides the means to establish a robust characterization of Martian organic (bio)geochemical processes. This dual focus on both abiotic components and potential biotic remnants represents a critical step in the search for extraterrestrial life within our Solar System.

## Background

### Martian habitability over time

The modern Martian surface is cold, dry, oxidizing, and perpetually bombarded with radiation, rendering it generally inhospitable to life *as we know it*. However, geological evidence suggests ancient Mars was more Earth-like and characterized by warmer temperatures, widespread fluvial activity, a thicker atmosphere, and a protective magnetic field shielding the surface from radiation^1^. Habitable conditions continued until cessation of the Martian dynamo terminated the planetary magnetic shield, leading to solar wind-induced atmospheric erosion and the loss of surface liquid water. Nevertheless, early environments inferred to be analogous to those found on Earth, common molecular feedstocks (including delivery of volatiles and organic matter by comets and asteroids), and plausible reactive pathways may have resulted in parallel origin of life events more than 3.4 billion years ago^2,3^.

### Organics as potential biosignatures on Mars

On Earth, life seemingly arose from a fortunate coalescence of chemistry and geology under placid conditions^3^. Was this also the case on ancient Mars? If so, did increasingly cold and dry conditions result in the extinction of Martian life, or did it adapt to the changing environment? Perhaps life never arose—if not, why? We believe these questions can be addressed with an organics-forward approach, because most of the building blocks of life form naturally through abiotic processes and are ubiquitous throughout the Solar System^4–7^. However, specific differences in their structures and distributions reveal crucial clues regarding their chemical origins^4,8,9^.

Terrestrial biology preferentially synthesizes a small and specific set of building blocks to create complex biopolymers. These include, for instance: a homochiral suite of 20 amino acids, phospholipids containing predominantly C_16_ and C_18_ straight-chain carboxylic acids, and five nucleobases along with ribose (also with chiral preference) to build nucleic acid informational polymers (*e.g*., DNA and RNA). In contrast, abiotic reactions within primitive accreting bodies (*e.g*., asteroids and comets) yield stochastically distributed molecular mixtures characterized by racemic amino acids, highly branched aliphatic carboxylic acids, hydrocarbons at low carbon numbers, and other molecular isomers not utilized by terrestrial biology. Furthermore, some of these organics can persist throughout geological timescales, with the potential to serve as evidence for either extant or extinct life^4,8,9^. On Earth, molecular fossils represent some of the most robust evidence of ancient life. If Mars experienced an independent genesis, similar organic remnants with structural preferences could persist in the geologic record today^3,4,10^.

### Mars exploration history

**Fig. 1:**
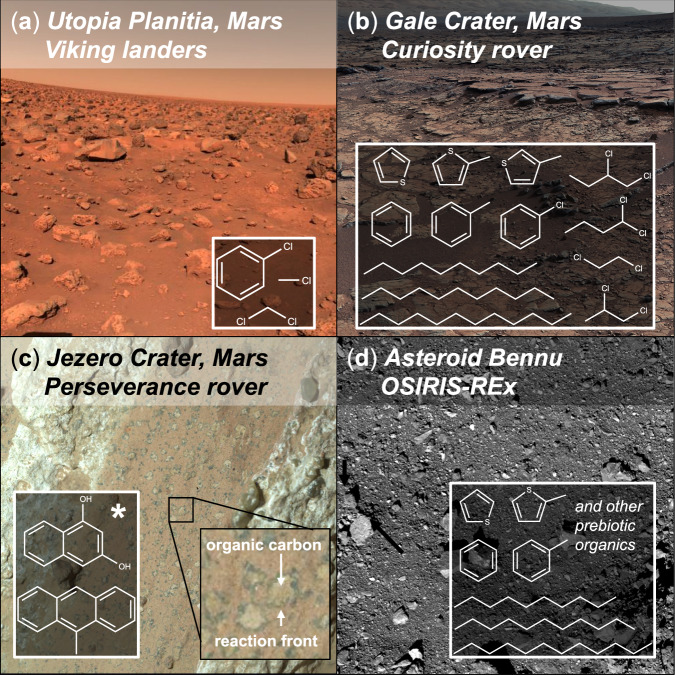
Organics have been detected on Mars by numerous landed missions carrying gas chromatography-mass spectrometry (GC-MS) and deep UV (DUV) Raman spectroscopy instruments. **a** The Viking 1 & 2 landers detected chlorinated breakdown products likely formed through interactions between organics and oxychlorine anions during thermal volatilization utilized for analysis^11,12^, **b** the Curiosity rover identified various functionalized organic fragments and long-chain *n*-alkanes (for simplicity, a subset is illustrated here)^13,15^, and the Perseverance rover measured redox-driven organo-mineral associations and polycyclic aromatic hydrocarbons (PAHs); * indicates that PAHs reported by Fornaro et al. (2025) were found in different locations and geologic units than organo-mineral associations reported by Hurowitz et al. (2025)^14,16^. **d** Carbonaceous meteorites and asteroids delivered to planetary surfaces contain a rich array of organics, including some of the same compounds detected on Mars (e.g., macromolecular carbon, thiophenes, PAHs, long-chain *n*-alkanes), along with a diverse suite of prebiotic organics, which highlights the importance of understanding abiotic background material when searching for organic biosignatures on Mars^5–7,18^. Image credits: **a** NASA/JPL, **b** NASA/JPL-Caltech/MSSS, **c** NASA/JPL-Caltech/MSSS, **d** NASA/Goddard/University of Arizona.

The search for life on Mars began with NASA’s Viking 1 and 2 landers (1976), which analyzed surface regolith in search of active biological metabolism and organic molecules with thermal volatilization-gas chromatography-mass spectrometry (TV-GC-MS) techniques. Although Viking did not find conclusive evidence for either, the 2008 discovery of oxychlorine salts by the Mars Phoenix lander indicated that perchlorate-driven oxidation during Viking thermal analyses (*i.e*., pyrolysis) likely obfuscated initial identification of indigenous organics^11^. Subsequent reanalysis of Viking data found trace chlorobenzene, likely produced through interactions between Martian organics and oxychlorines during pyrolytic thermal ramps^12^. Consequently, subsequent exploration strategies pivoted from direct life detection toward constraining paleo-habitability, aqueous geochemistry, and detection of preserved organic matter. In alignment with these objectives, NASA’s Mars Science Laboratory (MSL) and Perseverance rovers have since detected refractory organics, including possible exogenous meteoritic infall, in situ abiotic synthesis products, and complex organo-mineral associations (Fig. 1)^13–16^.

## Current objectives

Current astrobiological objectives for Mars encompass constraining past and present habitability over time, identifying bioavailable energy sources, elucidating mechanisms of organic synthesis, characterizing the abiotic-to-biotic chemical continuum, and searching for putative biosignatures^17^. Potential evidence for life includes macrostructures (e.g., body fossils), microtextures (e.g., stromatolites), mineral assemblages, chemical distributions, organics, and isotopes^17^. Of this set, molecular biosignatures detected via mass spectrometry techniques offer unparalleled diagnostic utility. In particular, specific organic compounds can suggest a biotic origin when their structural complexity or non-random distributions deviate significantly from stochastic abiotic distributions or expected thermodynamic equilibrium found in meteorites and returned asteroid samples. However, to serve as robust targets, these molecules must additionally display distributions indicative of structural utility or metabolic/enzymatic selectivity and demonstrate taphonomic preservation potential across geological timescales. Most importantly, they must be readily detectable by available flight or laboratory instrumentation^8^.

### Why organics?

Terrestrial biology synthesizes organic molecules through highly selective biosynthetic pathways. However, comparable suites of these essential compounds also form abiotically within primitive accreting bodies and the interstellar medium. A comprehensive inventory of prebiotic building blocks has been characterized in carbonaceous chondrites and returned asteroidal materials, including 15 of the 20 proteinogenic amino acids, vesicle-forming carboxylic acids (greater than eight carbons), and all components required for nucleic acid synthesis (nucleobases, sugars, and phosphate)^2,5–7^.

Because the structural preferences of biologically synthesized organics reflect specific cellular functions, complex molecules that deviate from the abiotic background may serve as robust indicators of extraterrestrial life. Key diagnostic features include: carboxylic acid or hydrocarbon chain lengths and branching patterns that deviate from meteoritic distributions, non-random structural isomers, restricted and homochiral suites of nucleobases, sugars, and amino acids, systematic isotopic fractionation, or preference for specific non-random isomers^2,4,8,9,18^.

## Outlook

### Returned sample analysis

Martian meteorites (Shergottites, Nakhlites, Chassignites) are the only Mars samples currently available for laboratory study today. These materials are predominantly young, igneous, lack geologic context, and can be easily compromised by terrestrial contamination, limiting their utility for trace organic analysis^19^. In recent years, sample-return missions from comets and carbonaceous asteroids have revolutionized our understanding of the early Solar System’s prebiotic inventory by leveraging state-of-the-art analytical instrumentation unconstrained by the mass, power, and environmental limitations inherent to in situ flight payloads^5–7^. In the coming decade, pristine material returned from Mars and its moon, Phobos, by CNSA’s Tianwen-3, JAXA’s Martian Moons eXploration (MMX) missions, and a possible NASA/ESA Mars Sample Return campaign, could enable the analysis of Martian organics using the same high-resolution analytical methodologies and contamination control techniques^6^.

### In situ sample analysis

To date, the Viking 1 and 2 landers remain the only Mars missions primarily dedicated to searching for direct molecular evidence of life. Five decades later, Viking’s analytical approach remains robust, demonstrated by MSL’s Sample Analysis at Mars (SAM) instrument, which detected organics with pyrolysis GC-MS. The forthcoming ESA ExoMars rover will build off SAM’s success, employing similar techniques with modernized instrumentation for the express goal of directly searching for extant or preserved molecular biosignatures^10^. Landed astrobiology missions carrying payloads optimized for biosignature detection can provide critical environmental context to complement and ground-truth sample return campaigns, access environments with high astrobiological potential, and provide additional datapoints from unique sites across Mars^8,10^. Priority targets include subsurface materials, sedimentary deposits, and subterranean ices, which mitigate the cumulative effects of ionizing radiation, and likely enhance the preservation potential of organic matter^4,10,20^.

### Characterizing organic geochemical processes on Mars

Here we propose that organics-based life detection on Mars requires a modernized analytical framework. A robust strategy must characterize organic geochemical processes from a planetary perspective, weighing abiotic inventories and putative biogenic remnants equally, to evaluate carbon within an abiotic-prebiotic-biotic continuum. This framework demands addressing fundamental unknowns. How do Martian organic geochemical processes compare to terrestrial ones? What is the composition of the present organic matter? Were these compounds synthesized in situ or exogenously delivered? What is the extent of their diagenetic alteration? This framework establishes results-agnostic objectives, yielding fundamental constraints on the Martian carbon cycle independent of the presence or absence of biology. Ultimately, characterizing Martian organics provides a critical baseline for constraining the synthesis and inventory of prebiotic organics in planetary environments, investigating our own molecular origins, and informing future life detection efforts across the Solar System.
